# MicroRNA-204 and microRNA-652-3p as promising biomarkers for endometrial cancer diagnosis and prognosis

**DOI:** 10.1590/1806-9282.20252283

**Published:** 2026-08-03

**Authors:** Yagmur Minareci, Hamdullah Sözen, Atahan Toyran, Canan Kucukgergin, Fatih Aydın, İlknur Bingul, Mehmet Guven Gunver, Serra Zeynep Akkoyunlu, Samet Topuz, Mehmet Yavuz Salihoglu

**Affiliations:** 1 Istanbul University, Istanbul Faculty of Medicine, Department of Gynecology and Obstetrics, Division of Gynecologic Oncology – Istanbul, Turkey.; 2 Istanbul University, Istanbul Faculty of Medicine, Department of Medical Biochemistry – Istanbul, Turkey.; 3 Istanbul University, Istanbul Faculty of Medicine, Department of Biostatistics – Istanbul, Turkey.

**Keywords:** MicroRNA, miRNA-204, Biomarkers, Endometrial cancer, Diagnosis, Prognosis

## Abstract

**OBJECTIVE:**

Endometrial cancer is the most common gynecologic malignancy in developed countries. Currently, there are no reliable screening methods available for the early diagnosis or prognostic evaluation of the disease. MicroRNAs are small non-coding ribonucleic acid molecules primarily involved in the regulation of gene expression. The aim of this study was to evaluate and compare serum levels of microRNA-204 and microRNA-652-3p between patients diagnosed with endometrial cancer and healthy controls in order to determine their potential diagnostic and prognostic value as candidate biomarkers.

**METHODS:**

Serum samples were collected from a total of 226 participants, comprising 150 patients diagnosed with endometrial cancer and 76 healthy controls. Relative expression levels of microRNA-204 and microRNA-652-3p were measured using quantitative real-time polymerase chain reaction.

**RESULTS:**

The present study demonstrated that serum levels of microRNA-204 and microRNA-652-3p were significantly elevated in patients with endometrial cancer compared with healthy controls. Overexpression of these microRNAs was significantly associated with disease stage and adverse prognosis. Moreover, patients with high microRNA-204 and microRNA-652-3p expression exhibited significantly shorter survival than those with low expression. Elevated expression of both microRNAs was also significantly associated with an increased risk of disease recurrence.

**CONCLUSION:**

Serum levels of microRNA-204 and microRNA-652-3p distinguished patients with endometrial cancer from healthy controls. In addition, both microRNAs were associated with prognosis, including an increased risk of disease recurrence. These findings require confirmation in larger, independently validated cohorts.

## INTRODUCTION

Endometrial cancer is the most common gynecological malignancy in developed countries, with a rising incidence worldwide^1^. Although early-stage disease has a 5-year survival rate above 80%, 15–20% of patients present with advanced-stage disease and poor-prognosis tumors for which current treatments rarely achieve a cure^2^. Early detection and better characterization of the molecular mechanisms driving endometrial tumorigenesis and progression are, therefore, essential to identify novel diagnostic and prognostic biomarkers and improve therapeutic strategies.

MicroRNAs (miRNAs) have emerged as promising molecules that may offer new insights into cancer diagnosis and treatment. These small, naturally occurring non-coding ribonucleic acid (RNA) molecules, typically 21–25 nucleotides in length, function primarily by regulating gene expression through partial complementarity with one or more messenger RNA ­molecules^3^. Aberrations or deficiencies in miRNAs that suppress oncogenes can contribute to cancer progression, highlighting their potential role in tumorigenesis^4^. Through their interactions with tumor suppressor genes or oncogenes, miRNAs actively participate in the complex process of cancer development^5^. MiRNAs can be detected not only in tissue but also in serum, urine, feces, sputum, saliva, tears, peritoneum, and cerebrospinal fluid samples^6^. Circulating serum miRNAs, in particular, have been proposed as candidate biomarkers for endometrial cancer diagnosis.

Previous studies have suggested an important role of miRNA-204 and miRNA-652-3p in endometrial carcinogenesis. Aberrant miRNA-204 expression has been reported in patient serum and has been linked to disease progression, while miRNA-652-3p has been associated with more aggressive tumor behavior and poorer outcomes. However, the diagnostic performance and prognostic value of circulating miRNA-204 and miRNA-652-3p remain incompletely defined^7-9^.

In the present study, we aimed to compare the serum ­levels of miRNA-204 and miRNA-652-3p between patients with endometrial cancer and healthy controls to assess their diagnostic and prognostic value as candidate biomarkers of the disease.

## METHODS

This prospective case-control study was conducted at Istanbul University Faculty of Medicine, Department of Gynecologic Oncology, in collaboration with the Department of Biochemistry. The institutional review board and ethics committee approved the study protocol (ethics number 1648/20, dated 11/2017). Written informed consent was obtained from all patients and controls. The primary endpoint was to investigate whether miRNA-204 and miRNA-652-3p could serve as potential circulatory biomarkers in patients with endometrial cancer. Overall survival (OS) was defined as the time from diagnosis to death from any cause; patients alive at last follow-up were censored. Disease-free survival (DFS) was defined as the time from surgery to the first recurrence/progression or death, whichever occurred first; patients without an event were censored at last follow-up. Survival outcomes were analyzed after stratifying endometrial cancer patients into high- and low-expression groups according to their miRNA levels.

### Study group

Eligible patients had histologically confirmed endometrial carcinoma of any stage according to the 2009 International Federation of Gynecology and Obstetrics (FIGO) classification, irrespective of histologic type or grade. Exclusion criteria were synchronous malignancies, uterine sarcomas other than carcinosarcoma, recurrent disease, and prior chemotherapy, radiotherapy, or targeted systemic therapy. Patients were followed every 3 months for the first 2 years, every 6 months from years 3–5, and annually thereafter. No patient received targeted therapies, including immunotherapy, as part of the initial treatment. Serum samples were obtained from all patients the day before surgery, before any treatment was initiated. Healthy controls were women attending the clinic for routine gynecologic care. All underwent gynecologic examination and transvaginal ultrasonography to exclude endometrial pathology. Controls with abnormal uterine bleeding, endometrial polyps, hyperplasia, or a history of hormonal medication use within the past 3 months were excluded. None had chronic inflammatory disease, diabetes, autoimmune disorders, or other systemic conditions known to affect circulating miRNA levels.

### Sampling and evaluation of microRNA-204 and microRNA-652-3p expressions

Peripheral venous blood was obtained from patients with endometrial cancer and from healthy controls at the Department of Gynecologic Oncology between November 2017 and December 2019. Samples were delivered to the Department of Biochemistry within 15 min of collection, centrifuged, and the serum fraction was separated. The serum was divided into aliquots and stored at -80 °C until analysis. Total RNA was isolated from serum using the miRNeasy Serum/Plasma Advanced Kit (Qiagen, USA; Cat. No. 217204) in accordance with the manufacturer's protocol. Reverse transcription was carried out with the miRCURY™ LNA RT Kit (Qiagen, USA; Cat. No. 339346) to generate cDNA. Quantification of miRNA-204 and miRNA-652-3p was performed by quantitative real-time polymerase chain reaction (PCR) on a Rotor-Gene Q platform (Qiagen) using miRCURY LNA™ miRNA PCR Assays for U6 (Cat. No. YP02119464), hsa-miR-204 (Cat. No. YP00206072), and hsa-miR-652-3p (Cat. No. YP00204387). Relative expression was calculated using the 2^-ΔΔCt^ method, with U6 serving as the endogenous reference control, as reported previously^9,10^.

### Statistical analyses

Statistical analyses were conducted using Statistical Package for the Social Sciences v20.0 and GraphPad Prism 5.0. For continuous variables that were not normally distributed, group comparisons were performed with the Mann-Whitney U test. Categorical data was analyzed using the χ^2^ test, or Fisher's exact test when appropriate. Survival outcomes were summarized with Kaplan-Meier estimates and compared between groups using the log-rank test. The diagnostic performance of each target miRNA was assessed by receiver operating characteristic (ROC) analysis; areas under the curve (AUCs) were calculated, and the optimal cut-off value was selected by maximizing the combined sensitivity and specificity. An AUC of 0.50 was interpreted as indicating no discriminative ability. Where applicable, effect estimates are reported as hazard ratios with 95%CIs. p<0.05 were considered statistically significant.

## RESULTS

A total of 226 women were included: 150 with endometrial cancer and 76 healthy controls. There were no significant ­differences in age, body mass index, parity, menopausal, or smoking status between endometrial cancer patients and healthy controls. Among cancer patients, the median age at diagnosis was 57 years (range 29–83), and most tumors were FIGO 2009 stage I and of endometrioid histology (Table 1). The median follow-up was 71.2 months (range: 1.2–95.7 months). Table 2 presents the associations between relative miRNA levels and clinicopathological characteristics in patients with endometrial cancer. Both miRNA-204 and miRNA-652-3p were significantly associated with tumor grade, nodal metastasis, depth of myometrial invasion, and disease recurrence, whereas miRNA-204 also correlated with lymphovascular space invasion (LVSI) and cervical involvement.

**Table 1:** Clinical characteristics of the endometrial cancer patients and healthy controls.

Parameter		Patient group (n=150)	Control group (n=76)	p-value
Median age, years (range)		57 (29–83)	55 (31–79)	0.881
Median BMI, kg/m^2^ (range)		32.0 (19.9–51.4)	32.1 (21.0–50.0)	0.801
Menopausal status, n (%)				
	Pre-/peri-menopausal	30 (25)	21 (27.6)	0.112
	Post-menopausal	120 (75)	55 (72.4)	
Smoking status, n (%)				
	Yes	34 (22.7)	29 (38.2)	0.061
	No	116 (77.3)	47 (61.8)	
Parity, n (%)				
	<1	32 (21.3)	18 (23.7)	0.372
	≥1	118 (78.7)	58 (76.3)	
Tumour histology, n (%)				
	Endometrioid	132 (88)	N/A	
	Serous	6 (4)	N/A	
	Carcinosarcoma	6 (4)	N/A	
	Mixed	5 (3.3)	N/A	
	Clear cell	1 (0.7)	N/A	
FIGO stage¶, n (%)				
	I	118 (78.7)	N/A	
	II	8 (5.3)	N/A	
	III	17 (11.3)	N/A	
	IV	7 (4.7)	N/A	

¶ According to FIGO 2009 classification for endometrial cancer, FIGO: international federation of gynecology and obstetrics; BMI: body mass index; OS: overall survival; DFS: disease-free survival; N/A: not applicable.

**Table 2:** Association between relative microRNA expression levels and clinical features in patients with endometrial cancer.

Parameter		n¥	Relative expression of miRNA-204¶	p-value	n	Relative expression of miRNA-652-3p¶	p-value
Age (years)							
	<50	30	1.335 (0.060–77.120)	0.912	30	1.300 (0060–9.410)	0.880
	≥50	119	1.480 (0.030–34.490)		120	1.380 (0.020–16.600)	
BMI (kg/m2)							
	<30	55	1.665 (0.020–77.120)	0.109	55	1.250 (0.060–9.410)	0.731
	≥30	94	1.160 (0.010–34.490)		95	1.280 (0.010–16.600)	
Tumor grade							
	1 and 2	112	1.900 (0.640–15.370)	<0.001	113	1.525 (0.330–9.410)	0.004
	3	37	6.010 (1.160–77.120)		37	1.950 (0.150–16.600)	
LVSI							
	Absent	113	1.970 (0.640–34.490)	0.011	114	1.460 (0.150–16.600)	0.073
	Present	36	6.190 (1.010–77.120)		36	2.170 (0.310–7.130)	
MELF type invasion							
	Absent	126	2.100 (0.640–34.490)	0.061	126	1.485 (0.150–16.600)	0.275
	Present	23	4.485 (1.030–77.120)		24	2.590 (1.170–6.160)	
Isthmic involvement							
	Absent	119	3.250 (0.640–34.490)	0.091	120	1.680 (0.270–18.900)	0.137
	Present	30	5.560 (1.070–97.110)		30	2.985 (1.314–6.740)	
Cervical involvement							
	Absent	133	1.900 (0.640–34.490)	<0.001	133	1.470 (0.150–16.600)	0.077
	Present	16	7.270 (2.970–77.120)		17	2.615 (1.614–6.160)	
Myometrial invasion							
	<50%	95	1.435 (0.640–139.630)	<0.001	96	1.360 (0.150–9.410)	<0.001
	≥50%	54	4.125 (0.970–3,289.140)		54	2.215 (1.220–161.400)	
Nodal metastasis							
	Absent	133	1.910 (0.640–34.490)	<0.001	134	1.470 (0.150–16.600)	<0.001
	Present	16	12.100 (2.130–77.120)		16	3.240 (1.614–7.130)	
Recurrence							
	Absent	116	1.895 (0.930–15.370)	<0.001	117	1.450 (0.150–7.130)	<0.001
	Present	33	7.035 (0.640–3,289.140)		33	2.410 (0.310–161.400)	

¶ Relative serum expression levels of miRNA-204 and miRNA-652 were pre­sented as median (range) in 2^-^ΔΔCT format

¥ miR-204 data were unavailable for one patient; BMI: body mass index; LVSI: lymphovascular space invasion; MELF: microcystic, elongated and fragmented pattern; miRNA: microRNA.

**Figure 1:**
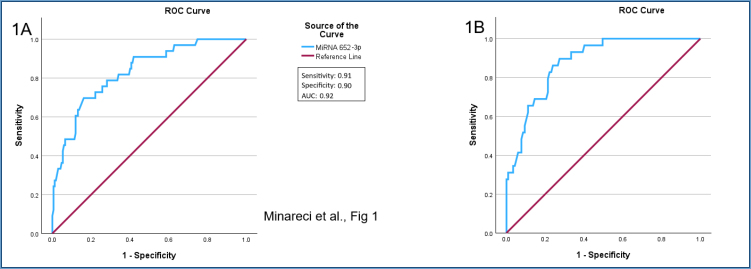
Receiver operating characteristic curve analysis of microRNA-204 (B) and microRNA-652-3p (A) as biomarkers of endometrial cancer. AUC: area under the curve.

Our study revealed that the serum miRNA-204 expression was 17.4-fold higher in endometrial cancer patients than in healthy controls (p<0.001). ROC analysis yielded an AUC of 0.95 (95%CI 0.92–0.97); at a cut-off value of 0.885, the relative sensitivity and specificity were 0.95 and 0.94, respectively (Figure 1).

Higher miRNA-204 levels were associated with more advanced FIGO stage, with significantly higher expression in stage III–IV than in stage I–II disease (p<0.001). OS and DFS differed significantly between high and low miRNA-204 expression groups (mean OS 24.05 vs. 65.1 months and mean DFS 12.66 vs. 63.4 months; both p<0.001). Patients who experienced recurrence had significantly higher median serum miRNA-204 levels than those without recurrence (p<0.001).

Serum miR-652-3p levels were 9.1-fold higher in patients with endometrial cancer than in healthy controls (p<0.001). A significant association was also identified between miRNA-652-3p overexpression and both disease stage and prognosis. ROC analysis yielded an AUC of 0.92 (95%CI 0.90–0.94); at a cut-off value of 0.510, sensitivity and specificity were 0.91 and 0.90, respectively (Figure 1). miRNA-652-3p expression increased with advancing FIGO stage (p<0.001). Patients with high miRNA-652-3p expression had significantly shorter overall and disease-free survival than those with low expression (mean OS: 54.7 vs. 69.3 months; p=0.017; mean DFS: 49.7 months vs. 61.6 months; p=0.003). Patients with recurrence had higher median serum miRNA-652-3p levels than those without recurrence (p<0.001).

In the subgroup analysis, we compared serum miRNA levels between patients with stage IA disease and healthy controls. In the stage IA subgroup (n=91), serum miRNA-204 levels were significantly higher than in controls, with an 8.53-fold increase (p<0.001). ROC analysis yielded an AUC of 0.87 (95%CI 0.81–0.90); at a cutoff of 1.045, sensitivity and specificity were 0.90 and 0.91, respectively. Although performance was attenuated relative to the all-stage cohort (AUC 0.87 vs. 0.95; fold change 8.53 vs. 17.4), these results indicate that circulating miRNA-204 retains clinically meaningful discriminative ability even at the earliest stage. Serum miRNA-652-3p levels were also higher in stage IA patients, with a 6.41-fold increase compared with controls (p<0.001) and an AUC of 0.83 (95%CI 0.80–0.86); at a cutoff of 6.05, sensitivity and specificity were 0.87 and 0.85, respectively. Although diagnostic performance was attenuated relative to the all-stage cohort (AUC 0.83 vs. 0.92; fold change 6.41 vs. 9.1), circulating miR-652-3p retained statistically significant discriminative ability in stage IA disease (p<0.001). Its performance nevertheless remained lower than that of miR-204.

## DISCUSSION

The identification of reliable, non-invasive biomarkers for early diagnosis and risk stratification is critical to improving clinical outcomes in patients with endometrial cancer. In this context, circulating miRNAs, which regulate key pathways involved in tumor proliferation and metastasis, have emerged as promising diagnostic and prognostic biomarkers in endometrial cancer^3,4^.

In the present study, we found significantly elevated serum expression levels of miRNA-204 in endometrial cancer patients. In addition, when the analysis was limited to patients with stage IA disease, the difference remained statistically significant. Similar to our results, Jia et al. found significantly higher miRNA-204 levels in 33 endometrial cancer patients than in 42 healthy controls, with an AUC of 0.74 (95%CI 0.594–0.885; p<0.001)^8^. In our cohort, miRNA-204 showed higher discriminatory ability, with an AUC of 0.95 (95%CI 0.92–0.97) for all stages and 0.87 (95%CI 0.81–0.90) in stage IA disease. Furthermore, in a recent systematic review conducted by Bloomfield et al., miRNA-204 was reported to be significantly downregulated in one study, whereas it was notably upregulated in two other studies comparing serum samples from endometrial cancer patients and healthy controls^11^.

According to our findings, elevated serum miRNA-204 levels were significantly associated with worse overall and disease-free survival. This clinical association, together with previous functional studies, supports a role for miRNA-204 dysregulation in pathways related to endometrial cancer progression. Chung et al. demonstrated that reduced miRNA-204 expression in endometrial cancer tissue modulates cell migration through FOXC1 and alters the expression of multiple genes involved in progression and metastasis^7^. Li et al. further highlighted a TrkB–STAT3–miRNA-204 regulatory circuit, in which TrkB-mediated activation of STAT3 suppresses miRNA-204 and enhances metastatic potential in endometrial carcinoma^12^. Although our serum-based results and these tissue-based data point in opposite directions with respect to miRNA-204 levels, they consistently indicate that disruption of miRNA-204–related pathways is linked to a more aggressive disease phenotype and suggest that tissue and circulating miRNA-204 may be differentially regulated.

In parallel with miRNA-204, serum miRNA-652-3p levels were higher in patients with endometrial cancer than in healthy controls, and this difference also persisted in the subgroup with stage IA disease. To the best of our knowledge, no clinical studies have yet evaluated serum miRNA-652-3p levels in patients with endometrial cancer. In our cohort, the diagnostic performance of serum miRNA-652-3p was slightly lower than that of miRNA-204, with an AUC of 0.92 (95%CI 0.90–0.94) for all stages and 0.83 (95%CI 0.80–0.86) for stage IA disease, compared with 0.95 and 0.87, respectively, for miRNA-204.

Furthermore, our findings indicate that higher serum miRNA-652-3p expression is associated with worse overall and disease-free survival and an increased risk of disease recurrence. These clinical data are in line with tissue-based studies. Sun et al. showed that miRNA-652 was significantly upregulated in endometrial cancer tissue compared with benign endometrium and that higher expression correlated with poor differentiation, recurrence, and worse prognosis, supporting its role as an onco-miRNA in endometrial cancer^9^. Consistent with a prognostic role, Chen et al. identified miRNA-652 as part of a five-miRNA tissue signature (miRNA-652, miRNA-3170, miRNA-195, miRNA-34a, and miRNA-934) that predicted 5-year survival in endometrial carcinoma with an AUC of 0.73^13^.

The present study evaluated the diagnostic accuracy and prognostic potential of serum miRNA-204 and miRNA-652-3p in patients with endometrial cancer, but several limitations should be noted. First, our cohort had a relatively limited number of patients; larger patient cohorts may yield more reliable results. Second, miRNA expression was analyzed solely in serum samples. Evaluating miRNA expression in endometrial tissue samples simultaneously might provide more accurate and robust results. Third, the prognostic significance of these biomarkers is inherently influenced by the cancer stage. Consequently, the prognostic value of these miRNAs cannot be conclusively determined without accounting for cancer stage. Finally, the diagnostic and prognostic values of circulating miRNA-204 and miRNA-652-3p have not yet been extensively validated in endometrial cancer. Therefore, further studies are warranted to confirm and expand upon these preliminary findings.

## CONCLUSION

Serum miRNA-204 and miRNA-652-3p levels were significantly higher in patients with endometrial cancer than in healthy controls, both in the overall cohort and in the stage IA subgroup, supporting their potential diagnostic utility. Higher serum ­levels of both miRNAs were also associated with poorer OS and DFS, and with an increased risk of recurrence, ­underscoring their prognostic relevance. These findings warrant external ­validation in larger, independent cohorts to confirm diagnostic and prognostic utility and to define clinically useful thresholds.

## Data Availability

The datasets generated and/or analyzed during the current study are available from the corresponding author upon reasonable request.
